# Anti-U1RNP-70kD-positive case of neonatal lupus presenting with seizure and incomplete heart block: a case report and literature review

**DOI:** 10.3389/fped.2023.1239327

**Published:** 2023-08-23

**Authors:** Maryam Alfalasi, Gehad ElGhazali, Waseem Fathalla, Khulood Khawaja

**Affiliations:** ^1^ Education Institute, Sheikh Khalifa Medical City (SKMC), Abu Dhabi, United Arab Emirates; ^2^ Sheikh Khalifa Medical City(SKMC), Purelab-Purehealth, Abu Dhabi, United Arab Emirates; ^3^ College of Medicine and Health Sciences, United Arab Emirates University (UAEU), Al Ain, United Arab Emirates; ^4^ Department of Pediatric Neurology, Sheikh Shakhbout Medical City (SSMC), Abu Dhabi, United Arab Emirates; ^5^ Department of Pediatric Rheumatology, Sheikh Shakhbout Medical City (SSMC), Abu Dhabi, United Arab Emirates

**Keywords:** neonatal lupus, systemic lupus erythematosus, heart block, seizure, case report

## Abstract

Neonatal lupus erythematosus (NLE) is an autoimmune disease caused by the transplacental passage of anti-Ro/SS-A and anti-La/SS-B. This can be less commonly seen with U1-ribonucleoprotein (U1RNP). Our patient is a 7-day-old male, who first presented with seizures. In addition, during an electroencephalogram, he was found to have an irregular heart rhythm. Looking further into the history, we found that the mother was aware that she had systemic lupus erythematosus (SLE). However, she had not been followed up with a rheumatologist. The workup for NLE found a negative anti-Ro/SS-A and anti-La/SS-B, with a positive U1RNP-70kD. U1RNP-70kD is a diagnostic test for mixed connective tissue disease in adults, but no research has been done on its significance in NLE. Despite having SLE, the infant’s mother did not receive surveillance during her pregnancy, as the current guidelines are tailored for mothers with anti-Ro/SS-A and anti-La/SS-B. As a result, this calls for the extension of these guidelines to include the U1RNP-70kD antibody. In this case, the 70kD subtype of U1RNP was positive, which may have had a role to play in this unusual presentation. However, further research is needed to improve the care of mothers and babies with U1RNP-70kD.

## 1. Introduction

Neonatal lupus erythematosus (NLE) is an autoimmune disease caused by the transplacental passage of pathological autoantibodies from the mother to the fetus. These antibodies are the anti-Ro/SS-A and anti-La/SS-B and less commonly the anti-U1-ribonucleoprotein (U1RNP) (1). Congenital heart block (CHB) occurs in 2% of mothers with anti-Ro/SS-A and/or anti-La/SS-B. This number increases up to 20% with subsequent pregnancies. The diagnosis for NLE involves the presence of one of the above antibodies in addition to a system involvement. It may present with multi-system involvement, including the skin, cardiac, hematologic, neurologic, and/or hepatobiliary system, usually manifesting as cutaneous lesions, cytopenia, elevated aminotransferases, and rarely heart block (1). Cutaneous manifestations have been associated with positive anti-U1RNP (1). Skin, hepatobiliary, and hematologic symptoms usually resolve within 6 months with the washout of maternal antibodies. On the other hand, the development of complete CHB is irreversible (2), with a mortality rate of 4%–29% (2). The cardiac involvement is not only limited to CHB but can also include structural abnormalities. In addition to CHB, the structural heart abnormalities carry a higher rate of mortality. Thus, detection and early intervention are required to decrease this risk of mortality (2). Here, we detail a case of NLE presenting with seizure and incomplete heart block, with positive anti-U1RNP-70kD and negative anti-Ro/SS-A and anti-La/SS-B.

## 2. Description of the case

This 7-day-old male was born full term, by a normal vaginal delivery. The mother was gravida 2, para 2. She was found to have group B streptococcus (GBS) and appropriately received antibiotics. She was also known to have hypothyroidism and was on levothyroxine but with an otherwise unremarkable perinatal history. The infant was brought to the emergency department (ED) with a cough and an increased difficulty in breathing. He was found to have respiratory syncytial virus (RSV) and was discharged with supportive management. The next day, he presented again to the ED with decreased activity and decreased oral intake. The mother reported symptoms of choking, coughing up frothy secretions, and experiencing weak extremities for a period of 4–5 min. A physical examination showed a hypoactive infant with normal suckling and Moro reflexes, an open anterior fontanelle, and no skin lesions. He was hemodynamically stable with no fever, weighing 2.6 kg (in the second percentile), with a length of 45 cm (less than second percentile), and a head circumference of 33 cm (in the fourth percentile).

**Figure 1:**
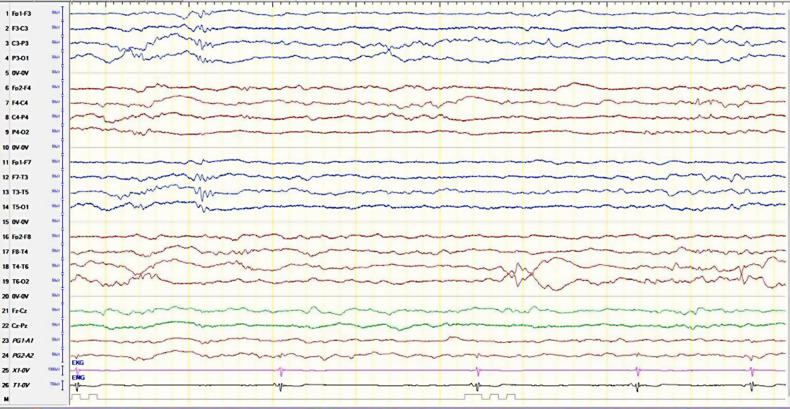
The electroencephalogram of the patient with the bradycardia/irregular heartbeat. Single-lead EKG monitor is in line 25.

The infant was admitted to the ward for observation, further management, and investigation. Full septic workup was sent, except for the lumbar puncture, which was refused by the parents. An electrocardiogram (ECG) and an electroencephalogram (EEG) were ordered. During the EEG session, the neurology physician noted an irregular heart rhythm on the ECG monitor, which was an evidence of an incomplete heart block (Figure 1). The extended history taken revealed that the mother had systemic lupus erythematosus (SLE), which, despite her being aware of it, was not previously revealed. She did not disclose this earlier as she did not think that it was of relevance because she was in remission, stopped taking her medication, and had not been followed up with a rheumatologist for over 18 months. This history alerted the team to investigate the infant for NLE. The neonate was then put on a Holter monitor and was sent to the pediatric intensive care unit (PICU) for close monitoring.

The EEG reported an underlying regional disturbance and a low threshold for seizures (the background was continuous with mixed frequencies and short epochs of discontinuity, in addition to frequent bilateral temporal sharp wave activity with no clinical activity). Subsequently, the patient was loaded with an intravenous (IV) levetiracetam at a dose of 30 mg/kg, with a maintenance dose of 15 mg/kg every 12 h. He was also started on empiric antibiotics, cefotaxime, and ampicillin for possible meningitis.

The detailed laboratory studies for the patient are presented in Table 1. In summary, his complete blood count and renal and liver function tests were within a normal range. His ammonia and glucose levels were normal. The anti-nuclear antibody (ANA) by indirect immunofluorescence (IIF) was 1:320 (positive speckled), complement 3 (C3) was low, complement 4 (C4) was normal, the extractable nuclear antigen (ENA) antibody test was positive, anti-Sm/RNP was detected by solid-phase assay, and anti-Sm antibody, anti-Ro/SS-A, and anti-La/SS-B were negative. The other autoantibodies were negative. His cardiac N-terminal prohormone of brain natriuretic peptide (NT-proBNP) and troponin T were high. His blood and urine cultures had no growth of any organism.

**Table 1:** Patient's investigations with reference and interpretations.

	Patient	Reference	Interpretation
White blood cells (×10^9^/L)	7.98	5.00–21.00	Normal
Red blood cells (×10^12^/L)	3.92	3.90–6.30	Normal
Hemoglobin (g/L)	127	125–205	Normal
Hematocrit	0.382	0.390–0.630	Low
MCV (fl)	97.4	86.0–124.0	Normal
MCH (pg)	32.4	31.0–37.0	Normal
MCHC (g/L)	332	305–355	Normal
Platelets (×10^9^/L)	337	140–400	Normal
Glucose (point of care) (mmol/L)	3.9	3.9–7.8	Normal
Sodium (mmol/L)	138	133–146	Normal
Potassium (mmol/L)	5.55	3.40–5.10	High
Chloride (mmol/L)	104	98–110	Normal
Bicarbonate (mmol/L)	24	22–29	Normal
Creatinine (μmol/L)	40	15–91	Normal
Urea (mmol/L)	3.37	2.80–8.10	Normal
Bilirubin total (μmol/L)	139.0		Normal
Bilirubin direct (μmol/L)	7.6	<10.0	Normal
Alkaline phosphatase (IU/L)	103	35–449	Normal
Aspartate transaminase (IU/L)	29	<50	Normal
Alanine transaminase (IU/L)	<5	<50	Normal
Ammonia (μmol/L)	48	16–60	Normal
NT-proBNP (pg/ml)	1,533		High
Troponin T (ng/L)	120		High
CRP (mg/L)	14.70	<5.00	High
ANA	1:320		High
C3 (g/L)	0.74	0.90–1.80	Low
C4 (g/L)	0.18	0.10–0.40	Normal
DNA Ab (DS) (IU/ml)	15.7	<26.9	Normal
ENA (CU)	164.9	<20.0	High
SmRNP	Positive		
Sm Ab	Negative		
Anti-U1RNP-70kD	Positive		
SS-A Ab	Negative		
SS-B Ab	Negative		
Ro-52 Ab	Negative		
DFS70	Negative		
DAT Poly	Negative		

Ab, antibody; ANA, anti-nuclear antibody; C3, complement 3; C4, complement 4; CRP, C-reactive protein; DAT, direct antiglobulin test; DFS70, anti-dense fine speckled 70; DsDNA, double-stranded deoxyribonucleic acid; ENA, extractable nuclear antigens; MCH, mean corpuscular hemoglobin; MCHC, mean corpuscular hemoglobin concentration; MCV, mean corpuscular volume; NT-proBNP, N-terminal prohormone of brain natriuretic peptide; SS-A, anti-Sjogren's syndrome A; SS-B, anti-Sjogren's syndrome B; U1RNP, U1-ribonucleoprotein.

As for the imaging, the echocardiogram showed a structurally normal heart with no signs of pulmonary hypertension. The cranial ultrasound showed a slightly increased periventricular echogenicity bilaterally, probably due to mild leukomalacia. No hemorrhage or hydrocephalus was observed. A brain computerized tomography (CT) angiogram was performed to exclude any vascular phenomena as a possible etiology for the seizure in the context of NLE, and it was found to be normal. The Holter monitor showed episodes of sudden sinus slowing, followed with junctional escape beats and periods of marked sinus bradycardia. He remained stable under observation in the PICU without requiring a pacemaker or any additional interventions. He was then discharged and put on oral levetiracetam at a dose of 40 mg twice per day.

Two weeks later, a follow-up in the clinic revealed no recurrence of seizures or development of new symptoms. Repeated laboratory workup showed improved cardiac function tests, with a decrease in ENA, but levels had not yet normalized. The EEG, ECG, and echocardiogram were normal. A month after discharge, a repeat EEG showed a normal result; therefore the levetiracetam was discontinued. With a normal physical exam, a follow-up at 6 months of age revealed that he was appropriately meeting his developmental milestones and the parents refused to repeat the laboratory workup at this visit.

Retrospectively looking at this case, although the mother did not disclose her history of SLE to the pediatric team, she was followed up with an obstetrics medicine physician who was aware of her SLE and immunology status. Since the mother lacked the classical anti-Ro/SS-A and anti-La/SS-B, this did not prompt the obstetrician to do further surveillance or education regarding the possibility of NLE. Table 2 presents the mother's immunology in early pregnancy.

**Table 2:** Mother's autoantibodies in early pregnancy.

	Mother	Reference	Interpretation
ANA	1:640		
C3	Not done		
C4	Not done		
Cardiolipin IgG (CU)	19.1	<20.0	Normal
Cardiolipin IgM (CU)	2.1	<20.0	Normal
DNA Ab (DS) (IU/ml)	97.3	<26.9	High
ENA (CU)	>429.4	<20.0	High
SmRNP	Positive		
Sm Ab	Negative		
Anti-Ro/SS-A	Negative		
Anti-La/SS-B	Negative		
Scl 70	Negative		
JO-1 Ab	Negative		

Ab, antibody; ANA, anti-nuclear antibody; C3, complement 3; C4, complement 4; DsDNA, double-stranded deoxyribonucleic acid; ENA, extractable nuclear antigens; SS-A, anti-Sjogren's Syndrome A; SS-B, anti-Sjogren's syndrome B.

## 3. Discussion

**Figure 2:**
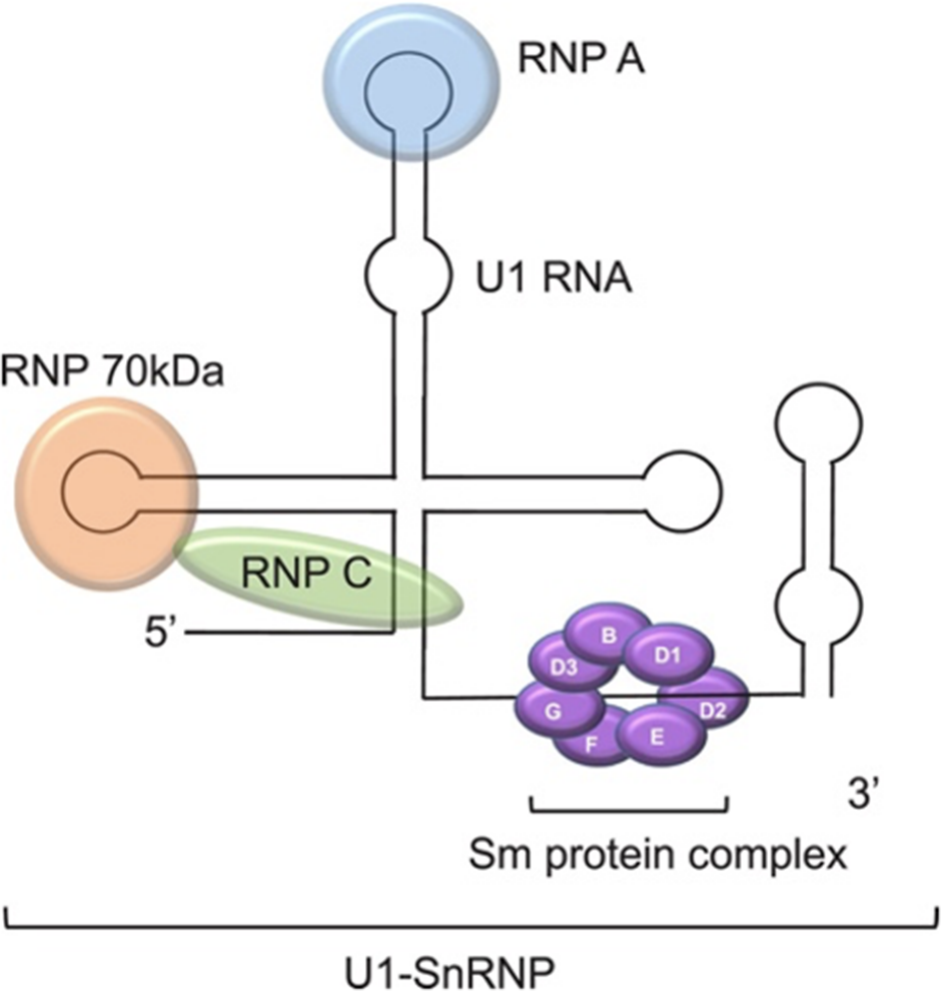
U1-SnRNP complex. Photograph obtained from an open-access article distributed under the Creative Commons Attribution License (3).

In this case, the interpretation of the immunology workup is important. Please note that this panel may appear differently in different labs. To summarize the immunology workup, ENA was high with negative anti-Ro/SS-A and anti-La/SS-B and positive Sm/RNP with negative anti-smith (anti-Sm) antibody. Sm/RNP is also known as U1-small nuclear RNP (U1-SnRNP) complex (Figure 2) (3). It is an ENA and is a complex protein consisting of both anti-Sm and anti-RNP. Both have their own subtypes. The protein subtypes of anti-RNP are 70kD, A, and C. U1RNP A and C are seen more in SLE patients, while U1RNP-70kD is specific for mixed connective tissue disease (MCTD) (4). In our lab, the anti-Sm is ordered as a separate entity, and it is negative. Hence, this concludes that the anti-RNP is positive. The U1RNP subtypes are not routinely tested as they pose no clinical significance or implication. We ordered it for our patients for future reference and for further studies. We have found that the U1RNP-70kD subtype is positive. No available data are reported on U1RNP subtypes in the literature in the context of NLE to compare this with.

Limited data on the clinical significance and pathogenicity of U1RNP subtypes in NLE were found; however some evidence from adult studies was outlined. Specifically looking at the central nervous system (CNS) involvement, the cerebrospinal fluid (CSF) of SLE and MCTD had been examined and found to have elevated indices of anti-U1RNP-70kD (5). Another study investigating the U1RNP in MCTD found that CNS involvement is higher in MCTD patients who have symptoms associated with SLE (6). The long-term follow-up of patients with MCTD found that 17% developed CNS involvement (7). Different subtypes of U1RNP may be associated with different clinical manifestations; therefore, we hypothesize that neonates of SLE mothers with U1RNP A/C present with cutaneous lesions, while neonates of SLE mothers with U1RNP-70Kd present with CHB and/or CNS involvement.

NLE rarely presents with neurologic manifestations. Few patients described in the literature that presented with neurologic symptoms, with the majority of cases finding CNS abnormalities in the imaging as a part of the NLE evaluation, were reported. Subependymal cysts, increased echogenicity, and hydrocephalus were the most widely seen radiologic changes. Table 3 summarizes the NLE patients in the literature with CNS manifestations (8–22). Psychiatric research and publications not in English are not included in this table. Development of seizure was reported in 10 patients (14, 16, 17, 19, 20, 22), eight of which had an underlying cerebral infarct or hemorrhage. The antibody profile is summarized in Table 3. One of the patients without a stroke is the most similar to our patient (14), but unfortunately, the U1RNP profile was not reported. The other symptomatic patients varied in presentation having myelopathy (8), spastic paraparesis (9), tetraplegia (15), facial nerve palsy with hemiparesis (18), phrenic nerve palsy (21), and hydrocephalus (10, 23). The majority of these neurologic symptoms resolved with no long-term sequelae.

**Table 3:** Literature review of neurologic involvement in NLE patients.

Year (reference)	Case	Neurologic manifestation	Neurologic symptoms or part of NLE evaluation?	Child antibodies		Other involvement
1987, Kaye et al. (8)	1	Lower limb spasticity, increased reflexes, and contracture at the ankle	Symptomatic	Anti-Ro/SS-A	Positive	Rash
				Anti-La/SS-B	Negative	
1993, Bourke et al. (9)	1	Spastic diplegia of lower limbs	Symptomatic	ANA Anti-Ro/SS-A Anti-La/SS-B	Positive	Rash, thrombocytopenia, transaminitis
1994, Nakayama-Furukawa et al. (10)	1	Hydrocephalus. CT head showed large ventricles and widening of extracerebral space	Symptomatic	ANA Anti-Ro/SS-A Anti-La/SS-B	Positive	Rash, annular erythema
				Anti-RNP Anti-Sm	Negative	
	2	Non-obstructive hydrocephalus. Ventriculoperitoneal shunt was placed	Symptomatic	Not done. Sister of the first case.		Rash, annular erythema
1996, Cabañas et al. (11)	1	Cerebral ultrasound scans showed hyperechogenic areas in the thalamus and/or basal ganglia (HTBG) indicating vasculopathy	Part of NLE evaluation	ANA Anti-Ro/SS-A Anti-La/SS-B	Positive	CHB, rash, thrombocytopenia
	2			ANA Anti-Ro/SS-A Anti-La/SS-B	Positive	CHB
	3			ANA Anti-Ro/SS-A	Positive	CHB
2003, Prendiville et al. (12)	1	Cerebral US: increased echogenicity of the white matter, subependymal cysts, and echogenic lenticulostriate vessels Brain CT: symmetrical, diffusely reduced attenuation of the cerebral white matter with normal attenuation of the gray matter	Part of NLE evaluation	ANA Anti-Ro/SS-A Anti-La/SS-B	Positive	Rash, hepatosplenomegaly with transaminitis, thrombocytopenia, irregular distal femoral metaphyses
	2	Cerebral US: Increased white matter echogenicity, bilateral subependymal cysts CT: Reduced attenuation of cerebral white matter, left subependymal hemorrhage and bilateral subependymal cysts	Part of NLE evaluation	Anti-Ro/SS-A	Positive	Rash, thrombocytopenia
	3	3 weeks of age Normal cerebral US Brain CT: Patchy peripheral reduced attenuation of cerebral white matter	Part of NLE evaluation	Anti-Ro/SS-A Anti-La/SS-B	Positive	Rash
	4	At birth Brain US: Mildly enlarged frontal horns of lateral ventricles Brain CT: Reduced attenuation of cerebral white matter, mildly enlarged frontal horns and third ventricle	Part of NLE evaluation	Anti-Ro/SS-A Anti-La/SS-B	Positive	Rash and thrombocytopenia
	5	At birth US: Increased white matter echogenicity, bilateral subependymal cysts, echogenic lenticulostriate vessels CT: Reduced attenuation of cerebral white matter, normal scan at 13 months of age	Part of NLE evaluation	Anti-Ro/SS-A Anti-La/SS-B	Positive	Hepatosplenomegaly with transaminitis and rash
	6	4 months of age Cerebral US and CT: Ventriculomegaly and increased cortical subarachnoid spaces	Part of NLE evaluation	ANA Anti-Ro/SS-A Anti-La/SS-B	Positive	Rash, hepatomegaly with transaminitis, hemorrhagic gastritis at 5 months
	7	2 months of age Brain CT: Bilateral basal ganglia calcification, ventriculomegaly	Part of NLE evaluation	ANA Anti-Ro/SS-A Anti-La/SS-B	Positive	Rash and neutropenia
	8	At birth Cerebral US: Bilateral subependymal cysts Brain CT: Reduced attenuation of cerebral white matter	Part of NLE evaluation	Anti-Ro/SS-A Anti-La/SS-B	Positive	Rash
	9	At 3 months, cerebral US and brain CT were normal	Part of NLE evaluation	ANA Anti-Ro/SS-A Anti-La/SS-B	Positive	Rash and neutropenia
	10	At 2 months, cerebral US was normal	Part of NLE evaluation	ANA Anti-Ro/SS-A Anti-La/SS-B	Positive	Rash and neutropenia
	11	At 2 months Cerebral US: Echogenic lenticulostriate vessels Brain CT: Bilateral basal ganglia calcification	Part of NLE evaluation	ANA Anti-Ro/SS-A Anti-La/SS-B	Positive	Rash and neutropenia
2004, Zuppa et al. (13)	1	Cerebral US: Subependymal pseudocyst	Part of NLE evaluation	Anti-Ro/SS-A 24	Positive	Thrombocytopenia
	2	Cerebral US: Subependymal pseudocyst	Part of NLE evaluation	Anti-Ro/SS-A 26	Positive	Thrombocytopenia, anemia, neutropenia, hepatic involvement
	3	Cerebral US: Normal	Part of NLE evaluation	Anti-Ro/SS-A 155 Anti-La/SS-B 126	Positive	Hepatic involvement
	4	Cerebral US: Bilateral subependymal pseudocyst	Part of NLE evaluation	Anti-Ro/SS-A 158 Anti-La/SS-B 154	Positive	None
	5	Cerebral US: Subependymal pseudocyst	Part of NLE evaluation	Anti-Ro/SS-A 160	Positive	None
	6	Cerebral US: Normal	Part of NLE evaluation	Anti-Ro/SS-A 194 Anti-La/SS-B 97	Positive	Hepatic involvement
	7	Cerebral US: Subependymal hemorrhage	Part of NLE evaluation	Anti-Ro/SS-A 209 Anti-La/SS-B 140	Positive	Hepatic involvement
	8	Cerebral US: Normal	Part of NLE evaluation	Anti-Ro/SS-A 187 Anti-La/SS-B 149	Positive	Anemia
	9	Cerebral US: Subependymal hemorrhage	Part of NLE evaluation	Anti-Ro/SS-A 203 Anti-La/SS-B 143	Positive	Hepatic involvement
	10	Cerebral US: Subependymal pseudocyst	Part of NLE evaluation	Anti-Ro/SS-A 305 Anti-La/SS-B 55	Positive	Neutropenia
	11	Cerebral US: Normal	Part of NLE evaluation	Anti-Ro/SS-A Anti-La/SS-B	Positive	CHB, anemia
2005, Lin et al. (14)	1	Two episodes of focal seizures. Brain ultrasonography: Normal ventricular size without a midline shift or intracranial or intraventricular hemorrhage. Brain CT showed generalized low density of the periventricular and deep white matter EEG: Rare spikes axial to the right parietal area	Symptomatic	ANA Anti-Ro/SS-A Anti-La/SS-B	Positive	Thrombocytopenia, rash, anemia
				Anti-DsDNA	Negative	
2012, Chen et al. (15)	1	Brain CT evidence for bilateral occipital hemorrhage. He was later diagnosed with tetraplegic cerebral palsy	Symptomatic	ANA Anti-Ro/SS-A	Positive	Thrombocytopenia and leukopenia
				Anti-La/SS-B Anti-Sm Anti-RNP	Negative	
2014, Saini et al. (16)	1	2-month-old girl with 5 days of fever, developed left-sided seizure. The focal seizure recurred on day 14 of illness. She had hypertonia and brisk muscle reflexes on the left side. MRI brain showed right hemispheric infarct with subdural hemorrhage. CT of head and neck vessels were consistent with vasculitis. At 15 months of age, she has mild motor delay with left hemiparesis	Symptomatic	ANA DsDNA	Negative	
2014, Döring et al. (17)	1	Focal seizure at day 1, confirmed by EEG. MRI brain revealed multiple ischemic areas in the distribution of the middle and posterior cerebral artery on the left side. Child fully recovered	Symptomatic	ANA Anti-U1-snRNP Anti-Sm	Positive	Rash, thrombocytopenia
				Antiphospholipid Anti-Scl 70 Anti-Ro/SS-A Anti-La/SS-B Anti-Tm Anti-Jo-1 Anti-DsDNA	Negative	
2014, Suthar et al. (18)	1	At age 6 months, she had CHB, but she remained asymptomatic until 2 years old when she presented with left-sided hemiparesis with left upper motor neuron facial nerve palsy MRI brain showed acute infarct in right putamen and internal capsule She had a pacemaker placed with residual weakness on the side of the hemiparesis	Symptomatic	Not tested		CHB
2016, Kanda et al. (19)	1	Seizures developed at day 2. Physical examination was normal. Brain imaging showed infarction of the left middle cerebral artery territory. Patient fully recovered with no sequelae	Symptomatic	ANA anti-Ro/SS-A	Positive	None
				Anti-La/SS-B	Negative	
2020, Wang et al. (20)	1	Seizure at 2 months. Brain MRI showed left middle cerebral artery stroke. Fully recovered by 3 years of age	Symptomatic	Anti-Ro/SS-A	Positive	None
	2	Developed global developmental delay at 6 months	Symptomatic	Anti-Ro/SS-A	Positive	CHB at 19 weeks of gestation
				Anti-La/SS-B	Negative	
2022, Paswal et al. (21)	1	Phrenic nerve palsy evident on chest x-ray with elevated diaphragm and confirmed with nerve conduction study	Symptomatic	Anti-Ro/SS-A Anti-La/SS-B	Positive	Anasarca, bradycardia, tachypnea, ascites, hepatomegaly, shock, CHB, thrombocytopenia, transaminitis
2023, Sun et al. (22)	1	Convulsions, EEG abnormalities	Symptomatic			Rash, heart
	2	Extracerebral space enlargement (giant cranium)				Rash, blood, liver, gastrointestinal, diabetic ketoacidosis
	3	Convulsions with subependymal hemorrhage	Symptomatic			Blood
	4	Hydrocephalus	Not clear			Gastrointestinal
	5	Subarachnoid hemorrhage	Not clear			Blood
	6	Subependymal hemorrhage	Not clear			Rash, blood, liver, gastrointestinal, heart
	7	Convulsions, hydrocephalus, periventricular–intraventricular hemorrhage, extracerebral space enlargement (brain atrophy)	Symptomatic			Rash, blood, liver, gastrointestinal
	8	Convulsions, hydrocephalus, subependymal hemorrhage, EEG abnormalities	Symptomatic			Blood, liver, heart
	9	Periventricular–intraventricular hemorrhage, extracerebral space enlargement (brain atrophy)	Not clear			Rash, blood, liver
	10	Convulsions, hydrocephalus, subarachnoid hemorrhage, EEG abnormalities	Symptomatic			Rash, blood, liver, gastrointestinal, heart, hypothyroidism

ANA, anti-nuclear antibody; CHB, congenital heart block; CT, computed tomography; DsDNA, double-stranded deoxyribonucleic acid; EEG, electroencephalogram; MRI, magnetic resonance imaging; NLE, neonatal lupus erythematosus; SS-A, anti-Sjogren's syndrome A; SS-B, anti-Sjogren's syndrome B; U1RNP, U1-ribonucleoprotein.

CHB is mostly seen with anti-Ro/SS-A and/or anti-La/SS-B. Only recently, CHB is being reported with anti-U1RNP with negative anti-Ro/SS-A and anti-La/SS-B (24). Similar to our case, a total of three patients who reported of having CHB with positive U1RNP (protein subtype not mentioned) and negative anti-Ro/SS-A and anti-La/SS-B but without any neurologic symptoms (24–26) were reported.

The current NLE recommendations for mothers with rheumatic diseases are targeted only for those with anti-Ro/SS-A and/or anti-La/SS-B antibodies. One of the recommendations is to use hydroxychloroquine during pregnancy (27). The other recommendation is early detection of CHB in the most vulnerable period of gestation. This is performed by measuring the mechanical PR interval with a weekly or bi-weekly Doppler fetal echocardiogram during 16–28 weeks of gestation (28).

It is important to rule out other possible causes or insults to diagnose NLE. In our case, GBS meningitis cannot be fully ruled out in the presence of a positive GBS result in the mother. In this admission, the mother received one dose of IV antibiotics, and the baby received a course of antibiotics. Our limitation here was the missing lumbar puncture. On the other hand, GBS meningitis was unlikely as it cannot explain the presence of heart block, and the patient remained well, afebrile with negative blood and urine cultures.

In summary, our patient had negative anti-Ro/SS-A and anti-La/SS-B and positive anti-U1RNP-70kD, presented with a seizure episode, and was found to have an incomplete heart block. The mother with SLE did not receive surveillance during pregnancy as the current guidelines are tailored for mothers with anti-Ro/SS-A and anti-La/SS-B. It is important to consider testing for U1RNP-70kD in newborns presenting with seizure and maternal SLE. In our case, the 70kD subtype of U1RNP is positive which may have a role in this unusual presentation. Further research looking at U1RNP and its subtypes in the context of NLE is needed to improve the care of mothers and babies with this antibody.

## Data Availability

The original contributions presented in the study are included in the article/Supplementary Material, further inquiries can be directed to the corresponding author.
